# Chemical constituents of tobacco smoke induce the production of interleukin-8 in human bronchial epithelium, 16HBE cells

**DOI:** 10.1186/s12971-016-0089-4

**Published:** 2016-07-19

**Authors:** Guojun Zhou, Weiqiang Xiao, Chengyun Xu, Yajun Hu, Xiaokai Wu, Fangfang Huang, Xinbo Lu, Chunyun Shi, Ximei Wu

**Affiliations:** Technological Center of China Tobacco Zhejiang Industrial Co., LTD., 118 Kehai Road, Hangzhou, Zhejiang Province 310024 China; Department of Pharmacology, School of Medicine, Zhejiang University, 866 Yuhangtang Road, Hangzhou, 310058 China

**Keywords:** Interleukin-8, Tobacco smoke, Human bronchial epithelium, Nicotine, Benzopyrene

## Abstract

**Background:**

Interleukin-8 (IL-8) functions as a major chemoattractant and plays pivotal roles in the initiation and development of chronic obstructive pulmonary disease (COPD), and tobacco smoke is a most risk factor contributing to the development of COPD. Hence, we have screened some of the tobacco smoke-derived chemical compounds that potentially induce the production of IL-8 in human bronchial epithelium, 16HBE cells.

**Methods:**

Twenty-eight hazardous smoke components belonging to 9 classes including nicotine, ammonia, aromatic amines, polycyclic aromatic hydrocarbons, phenols, carbonyls, hydrocyanic acid, nitrosamines and other volatile organics were used in the experiments. Proliferation of 16HBE cells was determined by cell counting kit-8 kit, luciferase activity was measured in *IL-8* reporter gene-expressing 16HBE cells, and IL-8 levels in culture supernatants were quantified by enzyme-linked immunosorbent assay.

**Results:**

At the non-toxic dosages, chemical compounds belonging to nicotine, aromatic amines, benzopyrene, phenols, aldehydes, and some other volatile organics dose-dependently increased *IL-8* reporter gene expression. Consistently, the representative compounds belonging to nicotine, aromatic amines, benzopyrene, phenols, aldehydes, and some other volatile organics significantly and dose-dependently increased IL-8 levels in the culture supernatants of 16HBE cells, among these compounds, benzopyrene is a most potent stimulator for inducing IL-8 production.

**Conclusions:**

The present study has identified particular tobacco smoke constituents responsible for inducing the IL-8 production in human bronchial epithelium, which might help shed light on the pathogenesis of tobacco smoke-induced COPD.

## Background

Tobacco smoke comprises a large amount of complex combustion compounds. It has been estimated that there are 7,357 chemical constituents, and of them, about 70 compounds have been confirmed with carcinogenic activity [1–3]. It is not feasible to measure all 7,357 components for product monitoring and subsequent regulation purposes. Therefore, a list of smoke components needs to be selected with a sufficiently broad chemical, pharmacological, and toxicological profile [4]. Hoffmann and his colleagues have published several lists with varying numbers of toxicologically and biologically active mainstream smoke components, which are well-acknowledged as Hoffmann analytes in both the tobacco industry and authorities [5–7]. In the list of Hoffmann analytes, 44 hazardous smoke components except for tar, nicotine, and carbon monoxide were divided into nine classes that are ammonia, aromatic amines, polycyclic aromatic hydrocarbons (PAH), phenols, carbonyls, hydrocyanic acid, nitrosamines, inorganic elements, and other volatile organics (Table 1).

**Table 1:** Chemical compounds used in this study and of Hoffman analytes

Category	Compounds	CAS NO	Category	Compounds	CAS NO
General compounds	Tar^*^	65996-92-1	Aromatic amines	1-Naphthylamine	134-32-7
	Nicotine	54-11-5		2-Naphthylamine	91-59-8
	Carbon monoxide (CO)^*^	630-08-0		3-aminobiphenyl	2243-47-2
Ammonia	Ammonia	7664-41-7		4-aminobiphenyl	92-67-1
PAH	Benzopyrene (BaP)	50-32-8	Carbonyl compounds	Methanal	50-00-0
Phenolic compounds	Phenol	108-95-2		Ethanal	64-17-5
	Catechol	120-80-9		Propanal	123-38-6
	Resorcinol	108-46-3		Butenal	4170-30-3
	Quinol	123-31-9		Acetone	67-64-1
	o-Cresol^*^	95-48-7		2-Butanone^*^	78-93-3
	m-Cresol	108-39-4		Propenal^*^	107-02-8
	p-Cresol	106-44-5		Butanal	123-72-8
HCN	Hydrocyanic acid (HCN)	74-90-8	Other volatile organics	Pyridine	110-86-1
Nitrosamines	N‘-Nitrosonornicotine (NNN)	16543-55-8		Quinoline	91-22-5
	Nitrosoanatabine (NAT)	887407-16-1		Styrene^*^	100-42-5
	N-Nitrosoanabasine (NAB)	1133-64-8		1,3-Butadiene^*^	106-99-0
	4-(N-Nitrosomethylamino)-1- (3-pyridyl)-1-butanone (NNK)	64091-91-4		Isopentadiene*	78-79-5
Inorganic elements	Ni, Pb^*^			Acrylonitrile*	107-13-1
	Cr, Cd^*^			Benzene	71-43-2
	As, Se, Hg^*^			Methylbenzene^*^	108-88-3

*These compounds were not included in this study due to their instability and unavailability. PAH, Polycyclic aromatic hydrocarbons

Though it is uncertain whether particular tobacco smoke constituents are responsible for specific adverse health outcomes, there is a broad scientific agreement that several classes of chemical compounds of burned tobacco are toxic and carcinogenic [8]. Tobacco smoke is a most risk factor contributing to the development of chronic obstructive pulmonary disease (COPD), a major cause of morbidity and mortality worldwide [9]. COPD is characterized by a slow and progressive decline in lung function and irreversible airflow obstruction, resulting from chronic airway inflammation, airway remodeling, and emphysema [10, 11]. Bronchial epithelial cells (BEC) not only form a first line of defense against inhaled chemical mixture of combustion compounds, but also initiate and control immune and inflammatory responses in COPD pathogenesis [12]. Upon activation by chemical mixture, BEC are activated and release pro-inflammatory cytokines and chemokines, which in turn recruit inflammatory cells including macrophages, neutrophils and dendritic cells. Activated immune cells further secrete inflammatory mediators, reactive oxygen species and proteolytic enzymes that contribute to the pathogenesis of COPD [13].

Among pro-inflammatory cytokines, interleukin-8 (IL-8, CXCL8), a member of the CXC chemokine family, plays a pivotal role in COPD pathogenesis [14, 15]. IL-8 is a major chemoattractant to neutrophils, which are responsible for inducing and sustaining the inflammatory state [16]. A close correlation between the number of neutrophils in induced sputum in COPD patients, the IL-8 level, and clinical outcome of the illness has been reported [17]. On the contrary, neutralization of IL-8 by a monoclonal antibody recognizing IL-8 in COPD causes a decrease of exacerbation frequency and clinical symptoms [18]. In the present study, we attempts to determine whether particular tobacco smoke constituents are responsible for inducing the IL-8 production in human normal bronchial epithelium, 16HBE cells.

## Methods

### Chemical compounds

Forty-four hazardous smoke components of Hoffmann analytes were listed, and 17 of them were not included in the present study due to their instability and unavailability (Table 1). Twenty-seven hazardous smoke components belonging to 9 classes were used in the present experiments. They are nicotine, ammonia, aromatic amines including 1-naphthylamine, 2-naphthylamine, 3-naphthylamine, and 4-naphthylamine, PAH including benzopyrene (BaP), phenols including phenol, catechol, resorcinol, quinol, m-cresol, and p-cresol, carbonyls including methanal, ethanal, propanal, butenal, acetone, and 2-butanone, hydrocyanic acid (HCN), nitrosamines including N’-Nitrosonornicotine (NNN), Nitrosoanatabine (NAT), N-Nitrosoanabasine (NAB), 4-(N-Nitrosomethylamino)-1-(3-pyridyl)-1-butanone (NNK), and other volatile organics including pyridine, quinoline, and benzene. All of these compounds were of analytic reagent and purchased from Sigma Co. (St. Louis, MO).

### Preparation of cigarette smoke extracts

Cigarette smoke extracts (CSE) were prepared according to a standard protocol as described previously [19]. Research-grade cigarettes (3R4F) were purchased from the Kentucky Tobacco Research Council (University of Kentucky, Lexington, KY). The cigarette components labeled on the packaging box of tobacco were as follows: total particulate matter, 10.9 mg/cigarette; tar, 9.4 mg/cigarette; and nicotine, 0.726 mg/cigarette. CSE was prepared by bubbling smoke from three cigarettes into 30 ml phosphate-buffered saline (PBS). CSE was standardized by measuring the absorbance at a wavelength of 320 nm. After filtered through a 0.45-mM filter, CSE were frozen in aliquots and stored at −80 °C. An aliquot of CSE was thawed immediately before use.

### Cell cultures

Human bronchial epithelial cells, 16HBE cells (generously provided by Professor Huahao Shen, Zhejiang University, Hangzhou, China), were grown in minimal essential medium with Earle’s salts (Life Technologies, Inc., Carlsbad, CA) supplemented with 10 % fetal calf serum (FCS, Life Technologies), 2 mM L-glutamine and 100 units/ml penicillin and streptomycin (Invitrogen, Grand Island, NY). The cells were subcultured before reaching confluence and incubated at 37 °C with 5 % CO2.

### Determination of cell proliferation by cell counting kit-8 kit

16HBE cells were seeded in 96-well plates at a density of 2 × 10^4^ cells/well, after pre-culture for 24 h, media were changed and the cells were treated with vehicle or indicated concentrations of chemical compounds for 72 h. After that, the cells were further incubated in the presence of counting kit-8 (CCK-8) solution (10 μM/well, Dojindo Molecular Technologies, Inc. Rockville, MD) for 4 h, and then subjected to absorbance determination by using a multiplate reader at the wavelength of 450 nm. The absorbance form cells treated with vehicle was defined as 100 %, and the absorbance from cells treated with tested compound was normalized to absorbance from cells treated with vehicle.

### Construction of IL-8 gene reporter plasmid

A complex human *IL-8* promoter region (nt −1475 to nt +1) was amplified by polymerase chain reaction (PCR) in the presence of genomic DNA from 16HBE cells as described previously [20, 21]. The 5’-GCACTCGAGTAACCCAGGCATT ATT-3’ and 5’- GCTAAGCTTAGTGCTCCGGTGGCTTTT-3’ were used as forward and reverse primers, respectively. The PCR product was further cloned into pGL6 reporter plasmid (Promega, Madison, WI) at *Kpn*I and *Xho*I sites to generate the luciferase reporter construct of *IL-8* (pGL6-IL-8-Luc), and the construct was verified by a DNA sequencer.

### Generation of 16HBE cells stably expressing IL-8 reporter gene

Stably IL-8 reporter gene-expressing 16HBE cells were generated as described previously [22, 23]. 16HBE cells were seed in a 6-well plate at a density of 1 × 10^5^ cells/well, after pre-culture for 24 h, cells were transfected with pGL6-IL-8-Luc for 24 h in the presence of serum. In each well, 4.8 μl Lipofectamin 3000 reagent, 8 μg pGL6-IL-8-Luc plasmid were used for transfection. After that, cells were selected in the growth medium containing G418 (800 μg/ml; Sigma) for 2 weeks. G418-selected colonies were picked up for expanding, and several clones that were positive for the introduced DNA were monitored by PCR. The clones with high response to 3 % CSE were chosen for the following experiments.

### Luciferase assay

16HBE cells stably expressing IL-8 reporter gene were plated in 24-well plates at a density of 8 × 10^4^ cells/well, after pre-culture for 24 h, cells were treated with vehicle or indicated concentrations of chemical compounds for 48 h. Then, cells were harvested, and the whole cell lysates were prepared in 100 μl reporter lysis buffer (Promega). The supernatants of cell lysates were used for protein quantification by Bradford assays (Beyotime Biotechnology, Shanghai, China) and luciferase assays according to the manufacturer’s instructions (Promega). The luciferase activity was normalized by protein level in the cell supernatants. The luciferase activities derived from vehicle-treated cells were defined as 1.

### Determination of IL-8 levels in culture supernatants by ELISA

16HBE cells were plated in 48-well plates at a density of 4 × 10^4^ cells/well, after pre-culture for 24 h, cells were treated with vehicle or indicated concentrations of chemical compounds for 48 h. Then, the cell supernatants were harvested for determination of IL-8 levels by human CXCL8/IL-8 quantikine ELISA Kit (R&D Systems Inc., Minneapolis, MN). The sensitivity of IL-8 assay is 7.5 pg/ml, and the inter- and intra-assay coefficients of variation for IL-8 measurements are 6.7 % and 4.6 %, respectively.

### Statistics

Numerical data were expressed as means ± SD and analyzed by one-way ANOVA and Tukey–Kramer multiple comparisons test. Differences were considered significant when *p* < 0.05 and *p* < 0.01. The SPSS statistical package (IBM, North Castle, NY, USA) was used for statistic assays. Experiments were independently repeated at least three times, the inter-experimental coefficients of variation were less than 10.6 %, and the representative results were shown.

## Results

### Proliferation of 16HBE cells after exposure to chemical compounds

**Fig. 1:**
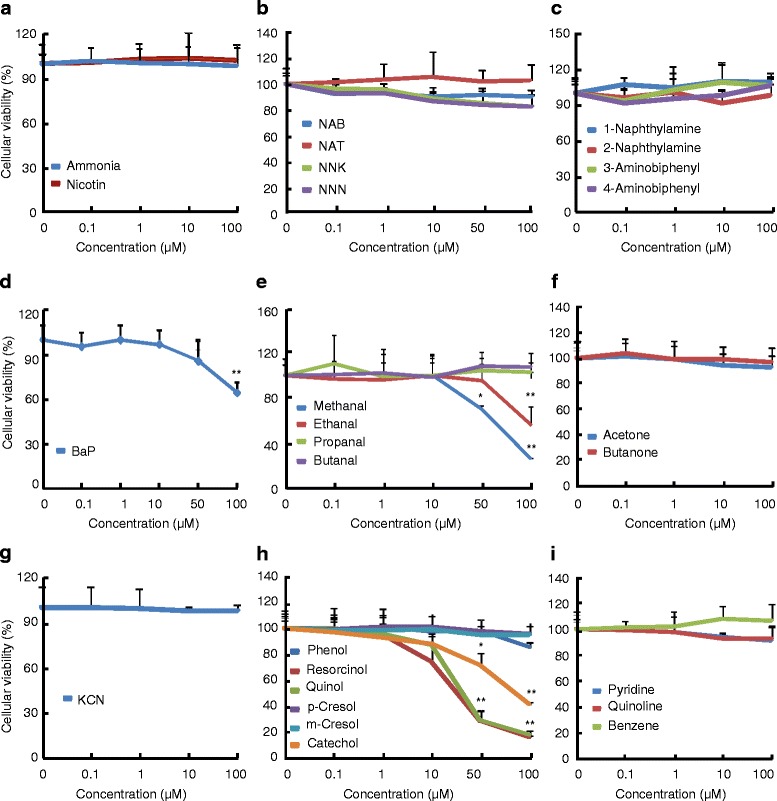
Proliferation of 16HBE cells in the presence or absence of chemical compounds. 16HBE cells were pre-cultured for 24 h, then treated with vehicle or indicated concentrations of chemical compounds for further 72 h. After that, cells were subjected to CCK8 proliferation assays. The data were expressed as means ± SD, *n* = 6. *p < 0.05, **p < 0.01 versus vehicle treatments (0 μM)

To investigate the potential toxicity of chemical compounds of tobacco smoke, we exposed the 16HBE cells to the compounds at concentrations ranging from 0 to 100 μM for 72 h, and performed the proliferation assays by CCK-8 kit. Treatments of cells with ammonia, nicotine, nitrosamines including NNN, NAT, NAB, NNK, aromatic amines including 1-naphthylamine, 2-naphthylamine, 3-naphthylamine, and 4-naphthylamine, carbonyls including propanal, butenal, acetone, and 2-butanone, HCN, phenols including phenol, m-cresol and p-cresol, other volatile organics including pyridine, quinoline and benzene led to no significantly inhibitory effects on the cell proliferation (Fig. 1a–i). However, treatment of cells with methanal at no less than 50 μM or ethanal and BaP at 100 μM resulted in significant decreases in the cell proliferation (Fig. 1d and e). Likewise, treatment of cells with resorcinol, quinol or catechol at no less than 50 μM caused significant decreases in the cell proliferation (Fig. 1h).

### Effects of chemical compounds on transactivation of *IL-8* gene

**Fig. 2:**
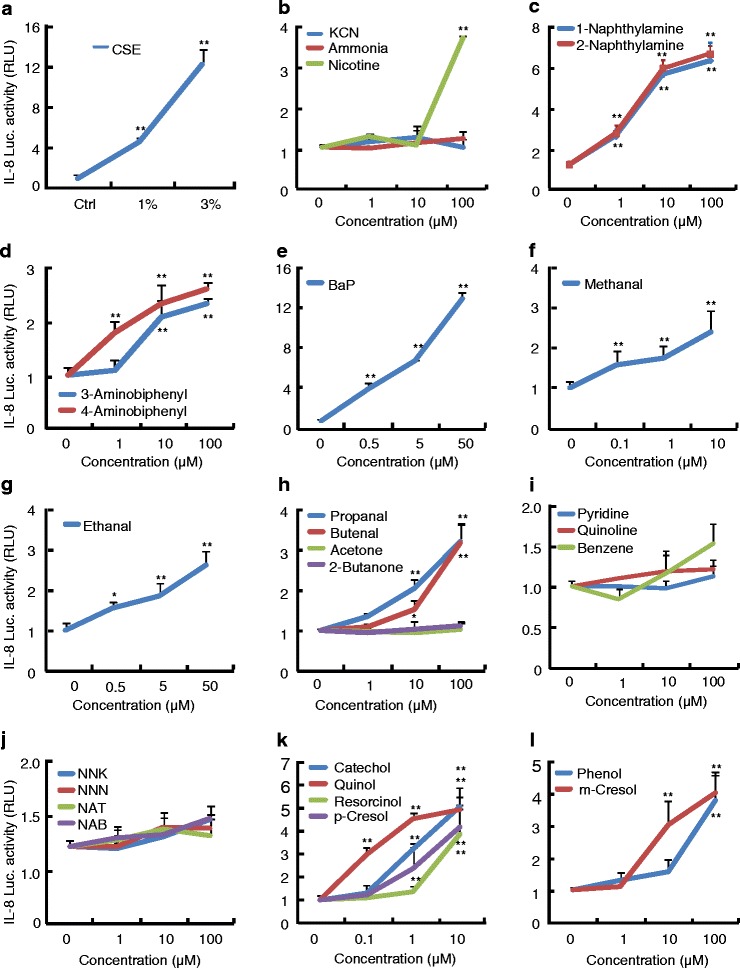
Luciferase activities in response to chemical compounds in 16HBE cells expressing IL-8 promoter-driven reporter gene. 16HBE cells stably expressing IL-8 promoter-driven reporter gene were pre-cultured for 24 h, then treated with vehicle or indicated concentrations of chemical compounds for further 48 h. After that, cells were harvested for preparation of whole cell lysates, the supernatants were subjected to luciferase assays and determination of protein levels. The data were expressed as means ± SD, *n* = 6. *p < 0.05, **p < 0.01 versus vehicle treatments (0 μM)

To investigate the potential effects of chemical compounds of tobacco smoke on transactivation of *IL-8* gene, we treated the 16HBE cells stably expressing IL-8 reporter with non-toxic dosages of chemical compounds for 48 h, and performed the luciferase assays. The 3 % CSE, a positive control treatment, led to approximately 11-fold increases in IL-8 reporter activities (Fig. 2a), nicotine ranging from 0 to 100 μM increased the IL-8 reporter activities up to approximately 3.5-fold (Fig. 2b), and aromatic amines including 1-naphthylamine, 2-naphthylamine, 3-naphthylamine, and 4-naphthylamine dose-dependently increased the IL-8 reporter activities, at 100 μM, they increased reporter activities by 2.0- to 6.0-fold over the control (Fig. 2c and d). However, neither ammonia and KCN nor nitrosamines including NAB, NNK, NNN, and NAT ranging from 0 to 100 μM had significant effects on the IL-8 reporter activities (Fig. 2b and j). Among the 27 compounds, Bap was the most potent agent in induction of reporter expression, it at 50 μM increased reporter activity by approximately 12.0-fold (Fig. 2e). Acetone, and butanone had no significant effects on reporter activity (Fig. 2h), whereas aldehydes including methanal, ethanal, propanal, and butanal induced reporter activities in dose-dependent manners, they at 100 μM increased reporter activities up to 2.0- to 3.0-fold (Fig. 2f–h). Moreover, all phenolic compounds induced the IL-8 reporter activities to different extents. Phenol, resorcinol, quinol, p-cresol, m-cresol, and catechol all at 100 μM increased the reporter activities by 2.7-, 4.2-, 6.3-, 3.4-, 3.0-, 3.8-fold, respectively (Fig. 2k and l). Benzene at different concentrations led to no significant changes in IL-8 reporter expression, whereas pyridine and quinoline both at 100 μM caused a slight increase in reporter expression (Fig. 2i).

### Effects of chemical compounds on the IL-8 levels in culture supernatants

**Fig. 3:**
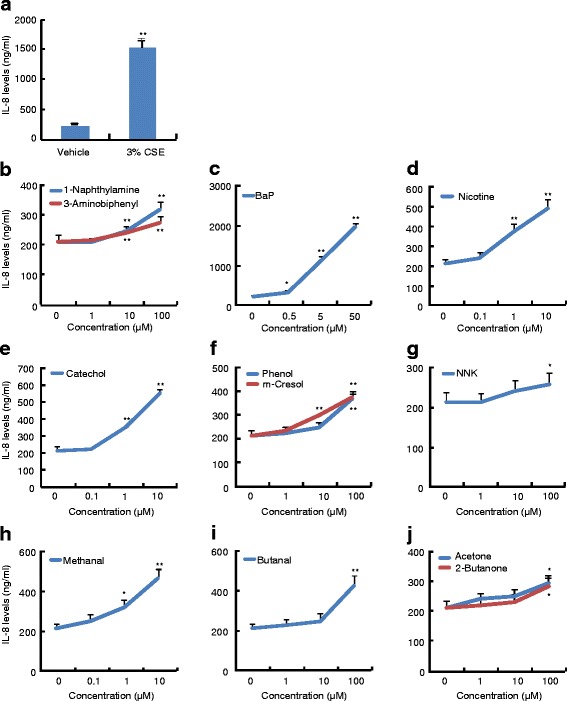
IL-8 levels in the culture supernatants of 16HBE cells. 16HBE cells were pre-cultured for 24 h, then treated with vehicle or indicated concentrations of chemical compounds for further 48 h. After that, the culture supernatants were harvested for determination of IL-8 levels by ELISA. The data were expressed as means ± SD, *n* = 6. *p < 0.05, **p < 0.01 versus vehicle treatments (0 μM)

To determine whether the chemical compounds that are able to induce IL-8 reporter expression increase IL-8 protein levels, we chose the representative compounds to treat cells for 48 h and determined the IL-8 levels in the culture supernatants by ELISA. Consistent with its induction of IL-8 reporter activity, the positive control treatment, 3 % CSE, increased the IL-8 levels by 7.0-fold in the culture supernatants (Fig. 3a). 1-naphthylamine and 3-amonobiphenyl used as the representatives of aromatic amines slightly induced the IL-8 levels, they at 100 μM increased IL-8 levels by 52 % and 33 %, respectively (Fig. 3b). Consistent with its robust induction of IL-8 reporter expression, BaP was the most potent one among these tested compounds that increased IL-8 protein levels, it at 50 μM increased IL-8 levels by 8.3-fold (Fig. 3c). Nicotine dose-dependently induced the IL-8 levels in the culture supernatants as well, and it at 100 μM increased IL-8 levels by approximately 3.1-fold (Fig. 3d). Phenol, m-cresol, and catechol used as the representatives of phenols, they at 100 μM induced the IL-8 levels in the culture supernatants by 71 %, 76 %, and 114 %, respectively (Fig. 3e and f). Methanal and butenal used as the representatives of aldehydes, methanal at 10 μM and butenal at 100 μM increased the IL-8 levels by 120 % and 125 %, respectively (Fig. 3h and i). Finally, NNK, acetone, and 2-butanone were used as the representatives of nitrosamines and carbonyls, respectively, all of them at 100 μM slightly increased IL-8 levels (Fig. 3g and j).

## Discussion

By using in vitro cell culture approaches, we have identified the tobacco smoke-derived chemical compounds capable of inducing the IL-8 production in human bronchial epithelial cells. Among 9 classes, nicotine, PAH, phenols, aromatic amines, aldehydes, and some other volatile organics induce the IL-8 production, and among 27 chemical compounds, BaP is most potent agent to induce the IL-8 production in 16HBE cells.

Thousands of chemical compounds exist in burned tobacco, it is not likely to identify all constituents responsible for stimulation of IL-8 production. We chose 44 hazardous smoke components that were listed in the well-acknowledged Hoffmann analytes [6, 7]. Because inorganic elements exist in combined but not free form in burned tobacco, they were not included in the present study. To screen the chemical compounds potentially inducing the IL-8 expression in human bronchial epithelial cells, we performed IL-8 promoter-driven luciferase assays followed by determination of IL-8 levels in culture supernatants. The cell’s sensitivity to CSE and likely to tested compounds depends upon the confluent state of cells, and cells in higher confluence are less frangible to CSE treatment [24]. Because we have plated cell at a same initial density and treated the cells with non-toxic dosages of chemical compounds for the same period (both of 48 h for reporter and ELISA assays), cells exposed to non-toxic dosages of compounds are supposed to be in the same density and the obtained results from the same type of assay are comparable. Moreover, since the cells respond robustly to the treatment of 3 % CSE, a positive control, in both IL-8 reporter assay and IL-8 protein determination, we suggest that our systems are applicable and robust for assessing the biological effects of chemical compounds from tobacco smoke.

Nicotine, PAH, phenols, aromatic amines, aldehydes, and some other volatile organics induce the IL-8 reporter activities, which well corresponds to their inducing the expression of IL-8 at protein levels, suggesting that increased IL-8 levels could be resulted from up-regulation of *IL-8* gene transcription. Induction of IL-8 production by aldehydes well corresponds to previous studies showing that unsaturated rather than saturated aldehydes induce the IL-8 production in human macrophages, small airway epithelial cells, and other human pulmonary cells [25–27]. However, which transcriptional factors are responsible for the transactivation of *IL-8* gene in response to these chemical compounds are still unknown. It has been reported that potential binding sites for transcription factors including interferon regulatory factor 1 (IRF-1), Hepatocyte nuclear factor 1 (HNF-1), activator protein 1 (AP-1), CCAAT/Enhancer Binding Protein (C/EBP), Nuclear factor-kB (NF-kB) present in the region of −133 kb to −85 kb of *IL-8* promoter, which is necessary for activation by some chemical compounds [25, 26]. Moreover, podechard et al. have found that BaP up-regulates IL-8 at mRNA and protein levels through aryl hydrocarbon receptor (AhR)-dependent transactivation of *IL-8* gene in primary human macrophages [27], corresponding to our findings showing that BaP is a most potent stimulator among the chemical compounds we have investigated. Thus, we suggest that the chemical compounds of tobacco smoke transactivate the *IL-8* gene and up-regulate the IL-8 expression possibly through activation of these transcriptional factors. Further experiments are required to address this issue.

Interestingly, all aromatic amines including 1-naphthylamine, 2-naphthylamine, 3-naphthylamine, and 4-naphthylamine led to significant induction of IL-8 reporter expression, all phenols including phenol, catechol, resorcinol, quinol, m-cresol, and p-cresol have significant effect on IL-8 reporter expression, and all aldehydes including methanal, ethanal, propanal, and butenal also robustly induce IL-8 reporter expression. On the contrary, all nitrosamines including NNN, NAT, NAB, and NNK have no obvious effects on IL-8 reporter expression. These findings support the notion that genre compounds possess the same property in induction of IL-8 reporter expression and IL-8 production. However, possibly due to no significant structural difference among the genre compounds, structure-activity relationship has not been found in our experiments. Though most of chemical compounds existing in tobacco smoke are at milligram, microgram, or nanogram levels that are significantly lower than dosages we used in the present study [6, 28], we suggest that the present study is still of great value according to the following reasons: (1) long-term exposure of chemical compounds to smokers results in their accumulation in bronchial epithelium and subsequent augmentation of their toxicity; (2) potential synergism among these compounds amplifies their toxicity; (3) unlike exposure of chemical compounds to the in vitro cultured cells in the form of solution, direct and successive inhalation of chemical compounds into bronchial epithelium of smokers possibly aggregates their toxicity. Thus, further experiments on the in vitro and in vivo effects of these chemical compounds are worthy of investigation.

Given the fact that tobacco smoke is a most risk factor contributing to the development of COPD, a major cause of morbidity and mortality worldwide [9], to identify the clinical significance of these hazardous compounds in the initiation and development of COPD is of great importance. Given the fact that decreases in inhalation of hazardous compounds benefit to not only smokers but also the people in polluted environments, our findings may have significant implications in specific adverse health outcomes.

## Conclusions

Taken together, the present study has identified several of the major classes of chemical mixture of burned tobacco responsible for inducing the IL-8 production, among 27 chemical compounds, BaP is one of most potent IL-8 inducer in human bronchial epithelium. Thus, the present study might help shed light on the pathogenesis of tobacco smoke-induced COPD.

## References

[1] Witschi H (2005). Carcinogenic activity of cigarette smoke gas phase and its modulation by beta-carotene and N-acetylcysteine. Toxicol Sci.

[2] Thielen A, Klus H, Muller L (2008). Tobacco smoke: unraveling a controversial subject. Exp Toxicol Pathol.

[3] Borgerding M, Klus H (2005). Analysis of complex mixtures--cigarette smoke. Exp Toxicol Pathol.

[4] Talhout R, Schulz T, Florek E, van Benthem J, Wester P, Opperhuizen A (2011). Hazardous compounds in tobacco smoke. Int J Environ Res Public Health.

[5] Djordjevic MV, Sigountos CW, Hoffmann D, Brunnemann KD, Kagan MR, Bush LP, Safaev RD, Belitsky GA, Zaridze D (1991). Assessment of major carcinogens and alkaloids in the tobacco and mainstream smoke of USSR cigarettes. Int J Cancer.

[6] Hoffmann D, Hoffmann I (1997). The changing cigarette, 1950–1995. J Toxicol Environ Health.

[7] Hoffmann D, Hoffmann I, El-Bayoumy K (2001). The less harmful cigarette: a controversial issue. a tribute to Ernst L. Wynder. Chem Res Toxicol.

[8] Witschi H, Espiritu I, Maronpot RR, Pinkerton KE, Jones AD (1997). The carcinogenic potential of the gas phase of environmental tobacco smoke. Carcinogenesis.

[9] Rabe KF (2007). Treating COPD--the TORCH trial, P values, and the Dodo. N Engl J Med.

[10] Vestbo J, Prescott E, Lange P (1996). Association of chronic mucus hypersecretion with FEV1 decline and chronic obstructive pulmonary disease morbidity. Copenhagen City Heart Study Group. Am J Respir Crit Care Med.

[11] Vestbo J, Lange P (2002). Can GOLD Stage 0 provide information of prognostic value in chronic obstructive pulmonary disease?. Am J Respir Crit Care Med.

[12] Gao W, Li L, Wang Y, Zhang S, Adcock IM, Barnes PJ, Huang M, Yao X (2015). Bronchial epithelial cells: The key effector cells in the pathogenesis of chronic obstructive pulmonary disease?. Respirology.

[13] Muller L, Jaspers I (2012). Epithelial cells, the “switchboard” of respiratory immune defense responses: effects of air pollutants. Swiss Med Wkly.

[14] de Moraes MR, da Costa AC, Correa Kde S, Junqueira-Kipnis AP, Rabahi MF (2014). Interleukin-6 and interleukin-8 blood levels’ poor association with the severity and clinical profile of ex-smokers with COPD. Int J Chron Obstruct Pulmon Dis.

[15] Liu HC, Lu MC, Lin YC, Wu TC, Hsu JY, Jan MS, Chen CM (2014). Differences in IL-8 in serum and exhaled breath condensate from patients with exacerbated COPD or asthma attacks. J Formos Med Assoc.

[16] Singer M, Sansonetti PJ (2004). IL-8 is a key chemokine regulating neutrophil recruitment in a new mouse model of Shigella-induced colitis. J Immunol.

[17] Celik H, Akpinar S, Karabulut H, Oktar P, Dursun B, Erguden HC, Gunay S, Sipit T (2015). Evaluation of IL-8 nasal lavage levels and the effects of nasal involvement on disease severity in patients with stable chronic obstructive pulmonary disease. Inflammation.

[18] Caramori G, Adcock IM, Di Stefano A, Chung KF (2014). Cytokine inhibition in the treatment of COPD. Int J Chron Obstruct Pulmon Dis.

[19] Chujo S, Okamoto S, Sunahara R, Adachi M, Yamada K, Hayashi H, Takii T, Hayakawa K, Onozaki K (2010). Cigarette smoke condensate extracts augment collagen-induced arthritis in mice. Int Immunopharmacol.

[20] Wu K, Katiyar S, Li A, Liu M, Ju X, Popov VM, Jiao X, Lisanti MP, Casola A, Pestell RG (2008). Dachshund inhibits oncogene-induced breast cancer cellular migration and invasion through suppression of interleukin-8. Proc Natl Acad Sci U S A.

[21] Tsuchiya K, Toyama K, Tsuprun V, Hamajima Y, Kim Y, Ondrey FG, Lin J (2007). Pneumococcal peptidoglycan-polysaccharides induce the expression of interleukin-8 in airway epithelial cells by way of nuclear factor-kappaB, nuclear factor interleukin-6, or activation protein-1 dependent mechanisms. Laryngoscope.

[22] Yin CH, Bach EA, Baeg GH (2011). Development of a high-throughput cell-based reporter assay for screening of JAK3 inhibitors. J Biomol Screen.

[23] Schwach G, Tschemmernegg M, Pfragner R, Ingolic E, Schreiner E, Windisch M (2010). Establishment of stably transfected rat neuronal cell lines expressing alpha-synuclein GFP fusion proteins. J Mol Neurosci.

[24] Heijink IH, Brandenburg SM, Postma DS, van Oosterhout AJ (2012). Cigarette smoke impairs airway epithelial barrier function and cell-cell contact recovery. Eur Respir J.

[25] Loftis JM, Morasco BJ, Hauser P (2011). Depression and antiviral response to interferon-based therapy for hepatitis C virus infection. Hepatology.

[26] Jamaluddin M, Choudhary S, Wang S, Casola A, Huda R, Garofalo RP, Ray S, Brasier AR (2005). Respiratory syncytial virus-inducible BCL-3 expression antagonizes the STAT/IRF and NF-kappaB signaling pathways by inducing histone deacetylase 1 recruitment to the interleukin-8 promoter. J Virol.

[27] Podechard N, Lecureur V, Le Ferrec E, Guenon I, Sparfel L, Gilot D, Gordon JR, Lagente V, Fardel O (2008). Interleukin-8 induction by the environmental contaminant benzo(a)pyrene is aryl hydrocarbon receptor-dependent and leads to lung inflammation. Toxicol Lett.

[28] Kopczynski SL (1989). Multidimensional gas chromatographic determination of cotinine as a marker compound for particulate-phase environmental tobacco smoke. J Chromatogr.

